# The characteristics of behaviour change interventions used among Pacific people: a systematic search and narrative synthesis

**DOI:** 10.1186/s12889-021-10420-9

**Published:** 2021-03-04

**Authors:** Amio Matenga-Ikihele, Judith McCool, Rosie Dobson, Fuafiva Fa’alau, Robyn Whittaker

**Affiliations:** 1 Epidemiology and Biostatistics, School of Population Health, University of Auckland, Private Bag 92019, Auckland, 1142 New Zealand; 2 Moana Research, Nga Hau Māngere Birthing Centre, 14 Waddon Place, Auckland, Māngere New Zealand; 3 National Institute for Health Innovation, University of Auckland, Auckland, New Zealand; 4 Pacific Health Section, School of Population Health, University of Auckland, Auckland, New Zealand

**Keywords:** Pacific health; health behaviour change, systematic review, talanoa

## Abstract

**Background:**

Pacific people living in New Zealand, Australia, United States, and the Pacific region continue to experience a disproportionately high burden of long-term conditions, making culturally contextualised behaviour change interventions a priority. The primary aim of this study was to describe the characteristics of behaviour change interventions designed to improve health and effect health behaviour change among Pacific people.

**Methods:**

Electronic searches were carried out on OVID Medline, PsycINFO, PubMed, Embase and SCOPUS databases (initial search January 2019 and updated in January 2020) for studies describing an intervention designed to change health behaviour(s) among Pacific people. Titles and abstracts of 5699 papers were screened; 201 papers were then independently assessed. A review of full text was carried out by three of the authors resulting in 208 being included in the final review. Twenty-seven studies were included, published in six countries between 1996 and 2020.

**Results:**

Important characteristics in the interventions included meaningful partnerships with Pacific communities using community-based participatory research and ensuring interventions were culturally anchored and centred on collectivism using family or social support. Most interventions used social cognitive theory, followed by popular behaviour change techniques instruction on how to perform a behaviour and social support (unspecified). Negotiating the spaces between Eurocentric behaviour change constructs and Pacific worldviews was simplified using Pacific facilitators and *talanoa.* This relational approach provided an essential link between academia and Pacific communities.

**Conclusions:**

This systematic search and narrative synthesis provides new and important insights into potential elements and components when designing behaviour change interventions for Pacific people. The paucity of literature available outside of the United States highlights further research is required to reflect Pacific communities living in New Zealand, Australia, and the Pacific region. Future research needs to invest in building research capacity within Pacific communities, centering self-determining research agendas and findings to be led and owned by Pacific communities.

**Supplementary Information:**

The online version contains supplementary material available at 10.1186/s12889-021-10420-9.

## Background

Pacific people experience a disproportionately high burden of long-term conditions and the associated risk factors [1–3], despite being minority communities in major countries such as the United States (USA), Australia, and New Zealand. Cardiovascular diseases, diabetes, cancer, and respiratory diseases represent a leading threat to human health and are the largest cause of premature mortality in the Pacific [4]. The burden of multi-morbidity is also more significant, with people experiencing two or more conditions requiring complex care and management [5]. These ongoing health inequities have persisted for several decades [6, 7] irrespective of large investments to improve Pacific health outcomes [3, 5].

There is a growing appreciation of how adopting lifestyle changes, such as being smoke-free, a healthy diet, regular physical activity, and limiting alcohol consumption, can positively affect health outcomes [8]. Even small changes in these health behaviours are known to have overall positive health gains [9]. The challenges when undertaking initial health behaviour changes and eventually maintenance recognises that identifying key characteristics that influence positive health change among Pacific people is necessary.

Behaviour change interventions often consist of multiple components designed to effect change in both an individual’s perceptions (cognitions) and behaviours [10]. These interventions are often underpinned by theoretical frameworks and may incorporate Behaviour change techniques (BCTs); methods identified as the ‘active ingredients’ of an intervention (e.g. goal setting) [11]. Behaviour change interventions that are grounded in theory are generally accepted to be more effective than those that lack a theoretical basis [10–12].

Health behaviour theories provide a framework for interventions designed to alter health-related cognitions and behaviours [12, 13]. Most theoretical frameworks used in public health and behaviour change interventions are predicated on the individual changing their behaviours [10]. These models, developed within Eurocentric perceptions of human behaviour, are built on the assumption that behaviour is ultimately individual and rational. This position dismisses other drivers to behaviour – namely, the spiritual, social, cultural, and environmental (collective) variables [12–14]. Social and cultural dynamics are also often sidelined within mainstream health behaviour change models. Understanding health behaviours and the context in which these occur is vital to designing effective interventions for minority and indigenous populations. Contemporary models of health behavior change are needed, which are inclusive inclusive, and reflect different culturally ascribed values, including wider spiritual, socio-cultural, and environmental influences [13] for Pacific people.

‘Pacific people’ is a broad, collective term used to describe the dynamic and diverse groups of people from the sub-regions of Oceania: Polynesia, Melanesia, and Micronesia. Distinct from a Western appreciation of health, health and wellbeing for Pacific people is holistic and intrinsically related to identity, land, cultural values, roles and responsibilities [15–17]. Although there are similar values observed within each Pacific Island, such as reciprocity, respect, relationships and collectivism, there are vast linguistic, cultural, geographical, migration and political differences between different Pacific Islands [16–18]. This diversity is further enhanced with place of birth (island born vs diaspora), and intermarriage where Pacific people now identify with two or more ethnic groups [14, 16, 17].

The family unit is an important institution for Pacific people, which extends beyond the nuclear family and is inclusive of village and church groups [19–22]. Aligning with the holistic Pacific *Fonofale* model [23], family remains an important foundation for Pacific people, providing a stable system of support despite social and economic changes, transnational migration, urbanisation and modernisation [15, 24]. An individual’s motivation to change behaviour is strongly interwoven with the socio-cultural roles and family responsibilities [20, 25–27]. Several studies have highlighted Pacific people to perceive health as being able to provide for their families and the wellbeing of their family unit, rather than the physical ailments of an individual [21, 28, 29]. Moreover, studies that have included family and social support have shown to be more effective than those requiring individuals to undertake behaviour change on their own accord [30]. Understanding the determinants of behaviour change for Pacific people is a necessary step towards designing interventions that are culturally relevant, salient and sustainable [14].

Acknowledging the cultural differences, values, beliefs, structures, practices and worldviews of health and wellbeing unique to Pacific people can provide the contextual framework for developing health behaviour interventions [25, 28]. Fundamental to the success of any programme centres around creating interventions that are acceptable and accessible for Pacific people, who have “the right to the highest attainable standard of health” in New Zealand [31]. Evidence suggests that traditional or non-Pacific programmes have not been effective, despite considerable investments [22, 27, 32]. Furthermore, it is important to consider the role of inadequate workforce capability in cultural diversity, institutional racism, and unconscious bias have contributed to the state of Pacific health in New Zealand [3, 15, 22, 27, 33].

Pacific led programmes reflect the importance of relational rather than individualistic relationships among Pacific people. The significance of relationships can be understood using the Samoan concept of *‘teu le va’,* which is premised heavily on the relational contexts between people, things and the environment, as well as the nurturing and protection of mutually respectful relationships over individual agendas [15, 34, 35]. The culturally located concept of ‘*va*’ (relationships) cannot be measured and is not inherently visible within western frameworks. When this ‘space’ is appropriately nurtured, respect and trust ensue, translating into a higher acceptance of programme initiatives and health care [15]. It is also acknowledged in practical terms, such as when health workers or providers are from the same ethnicity or cultural background, interactions are reciprocated with respect, and better health outcomes are achieved [14, 22, 27].

Community inspired behaviour change requires the adoption of Community Based Participatory Research (CBPR) methods, a collaborative approach used in public health with minority communities [10, 36]. True to the CBPR methodology, community members, organisations, and researchers become equal partners in all aspects of the research process [36], addressing health from a holistic perspective. It builds trust among those who may have had negative experiences, and distrust researchers and the research process [10]. CBPR also aligns with several core Pacific principles: reciprocity, nurturing relationships, respect, collectivism, and communitarianism [16, 17]. Valuing the partnerships between academia, health professionals and communities through CBPR has the potential to design and facilitate health behaviour change within the socio-cultural context of communities [10]. Current Pacific health research guidelines [16] emphasise the need for such approaches, which aligns with indigenous guidelines developed for Māori in New Zealand [37], Aboriginal and Torres Strait Islanders in Australia [38] and First Nations communities in Canada [39].

Improving the health and wellbeing for Pacific people is an important equity issue [3, 15, 28]. Engaging meaningfully and negotiating the spaces between Eurocentric theoretical frameworks and Pacific cultural constructs of health and wellbeing [40] is needed if evidence-based and culturally contextualised behaviour change interventions are to be adopted and sustained. The aim of this systematic search and narrative synthesis is to describe the behaviour change components used in interventions designed to improve health and effect health behaviour change among Pacific people.

## Methods

The systematic review was informed by the Preferred Reporting Items for Systematic Reviews and Meta-Analyses (PRISMA) reporting guidelines [41].

### Eligibility criteria

Studies were eligible to be included if they described an intervention designed to change health behaviour(s) in Pacific people. Included studies were required to report a clinical or behaviour change outcome with or without randomised controlled conditions. Studies were also required to have at least 40% of participants identifying with a Pacific ethnicity and include participants aged over 16 years. Studies were excluded if they were not published in the English language or the full text was not available. There was no time period restriction applied to the search.

### Information sources

The OVID Medline, PsycINFO, PubMed, Embase and SCOPUS databases were searched during January and February 2019, and again in January 2020 by the first author (AI). Additional articles were identified from reference lists. A manual search of the New Zealand Medical Journal, Pacific Health Dialog, Pacific Health Review, Fiji Journal of Public Health and the Australian and New Zealand Journal of Public Health was also conducted during March 2019 and January 2020 for further articles that may not have been included during the initial database searches.

### Search strategy

The search strategy used in Medline is provided in Table 1. The keywords, their synonyms and various spellings were reviewed and agreed upon by three co-authors prior to commencement. Terms used included 1) Pacific population groups and phrases related to 2) health behaviour change. Search terms were entered according to each database’s requirements and using the Boolean operator of and/or to combine terms. Database specific filters such as human population, English language, and full-text articles were applied where available.

**Table 1:**
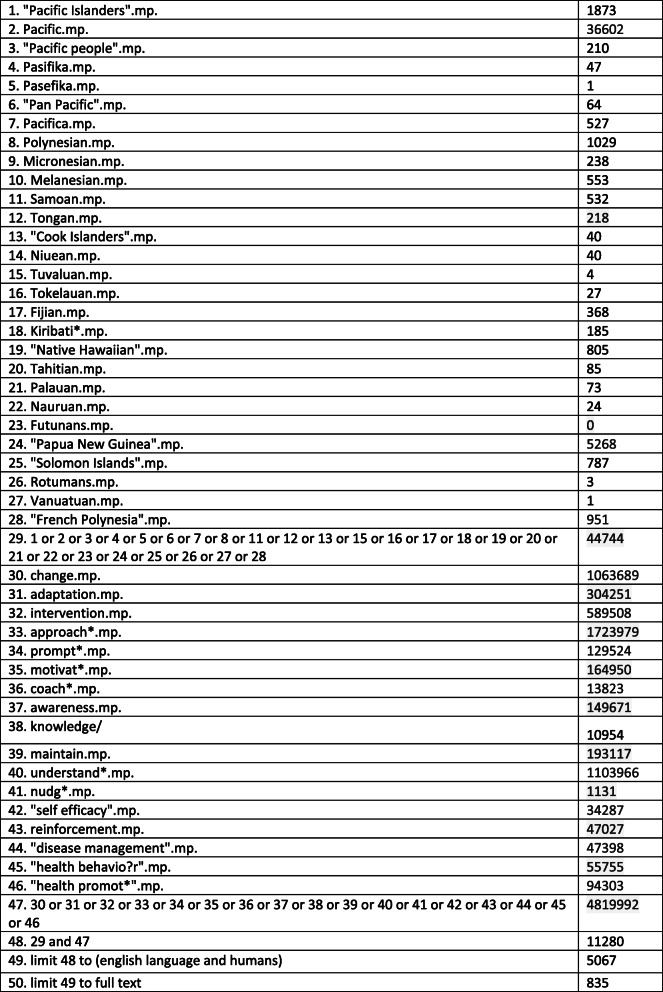
OVID Medline search strategy

### Study selection

All articles were downloaded into RefWorks ProQuest software, and duplicates were removed. Articles were screened for eligibility based on their title and abstracts by the first author (AI) and sorted accordingly. Full-text articles were then retrieved for the remaining papers and non-relevant studies excluded based on the inclusion criteria. The quality of studies were appraised using the Joanna Briggs Institute (JBI) Critical Appraisal Checklist [42], a quality assessment tool to determine the methodological quality of quantitative studies. Studies were appraised by the first author (AI) and crosschecked by two co-authors (JM and RD) to determine the final decision, and a consensus on inclusion or exclusion reached.

### Data extraction

Using the PRISMA checklist [41], study information including study characteristics, participant information, outcomes and behaviour change theoretical frameworks were extracted and included in a Microsoft Excel spreadsheet. Study characteristics included authors, year of publication, targeted health condition, the country in which the study was conducted, and sample sizes. Participant information included age, gender, and ethnicity.

For each study BCTs were identified and coded using the behaviour change taxonomy [11]. The 93 BCTs were rated as either present (1) or absent (0). BCTs were coded when there was clear evidence of inclusion despite the wide-ranging terminology used. For instance, when the interventions mentioned education around “discussing complications” or “reducing risk”, it was coded as information on health consequences (5.1). Social support (general) (3.1) was also coded as such, even when it was not always explicitly mentioned. For example, if studies required family members or a significant other to be part of the intervention, or the studies that delivered group sessions emphasised group support as an important component for behaviour change. When BCTs were not explicitly described as part of the intervention, codes were obtained from curriculum lesson outlines where available.

## Results

**Fig. 1:**
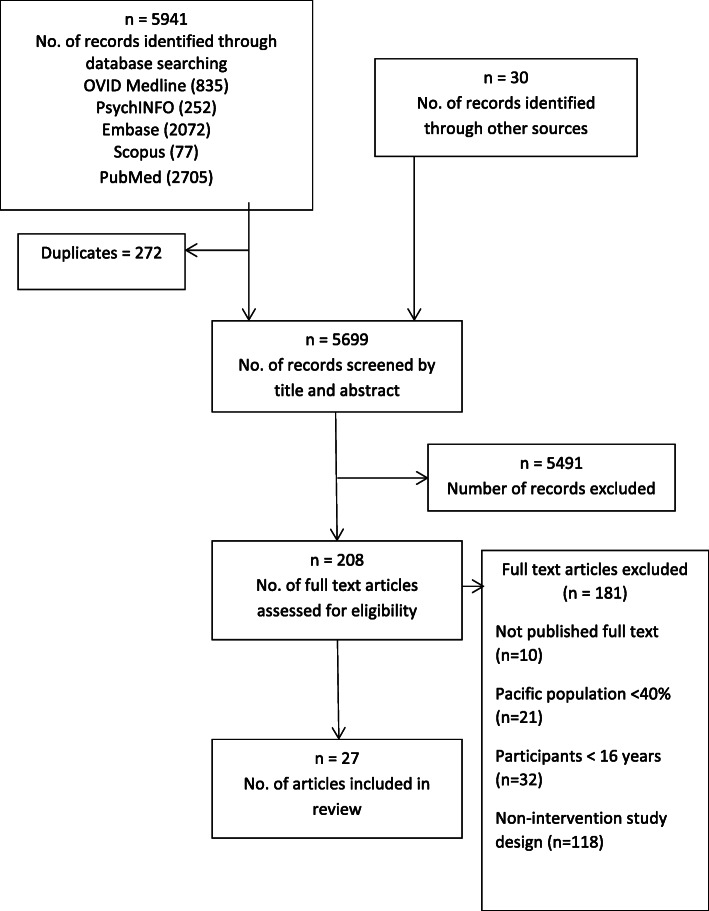
PRISMA study selection process

A total of 5941 records were identified using the search strategy described. An additional 30 articles were identified by searching references. After removing duplicates, 5699 articles were reviewed by title and abstract of which 5461 were excluded. The full text of 208 articles were reviewed with articles excluded if they were not an intervention study (*n* = 132), studies focusing on participants aged under 16 years (*n* = 39), less than 40% of participants identified as Pacific (*n* = 27) and full-text articles not available (*n* = 10). Twenty-seven articles met the inclusion criteria and are included in this review. Figure 1 shows the study selection process. Five of the included studies reported on the same parent intervention - the *Pili Ohana Project* (POP). These studies are included separately as they each provide unique data and use different cohort samples and intervention settings.

### Study characteristics

Fifteen studies were conducted in the USA - Hawai’i [43–55], California [56] and Arkansas [57], as well as its associated territories of American Samoa [58, 59] and the Marshall Islands [60]. Seven were based in New Zealand [61–67] including one in Australia [68] and one in Samoa [69]. Most studies involved Native Hawaiians residing in the state of Hawai’i. Nine studies [43, 44, 46–48, 50–52, 64] used a multiethnic cohort comparing Pacific (Native Hawaiians, Samoan, Tongan, Chuukese), Asian, Caucasian or other non-Pacific population groups. Seven studies focused on Pacific only cohorts, such as Native Hawaiian and other Pacific Islanders [45, 53, 55, 56], Tongan and Samoan [62] and, Tongan, Niue and Cook Island [61] and Pacific Islanders (mixed) [67]. Two studies compared Pacific and Indigenous Māori [65, 66]. Nine studies were ethnic-specific focusing only on Native Hawaiians [49, 51], American Samoan [58, 59], Marshallese [57, 60], and Samoan [63, 68, 69] populations. Studies were published between 1996 and 2020. Table 2 shows further details of the included studies.

**Table 2:**
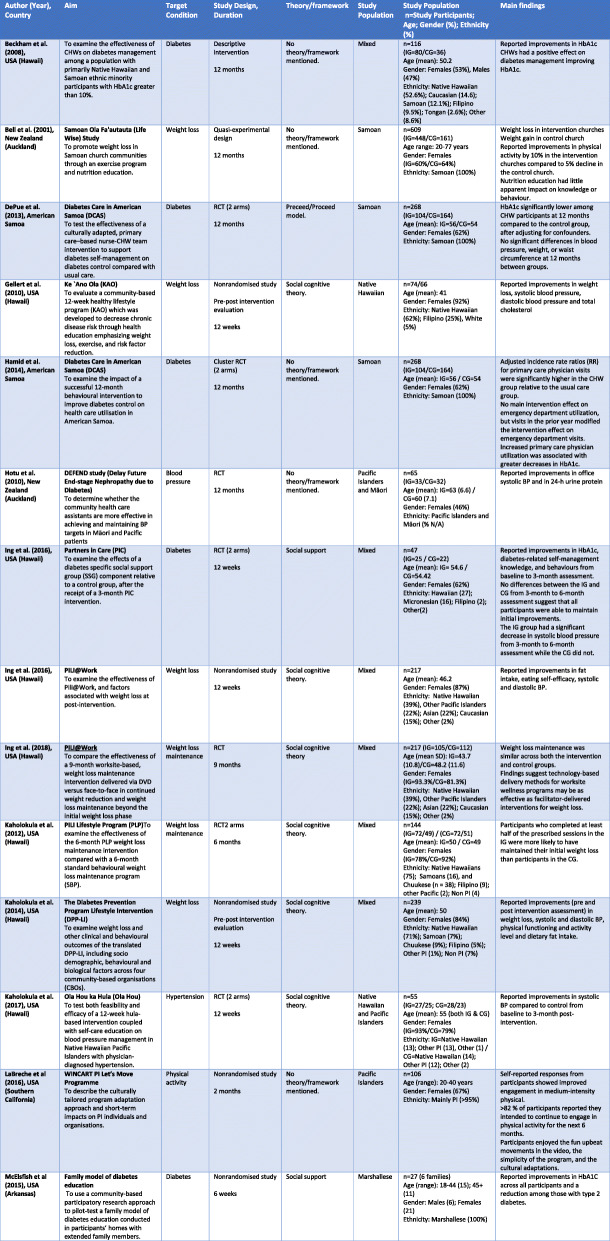

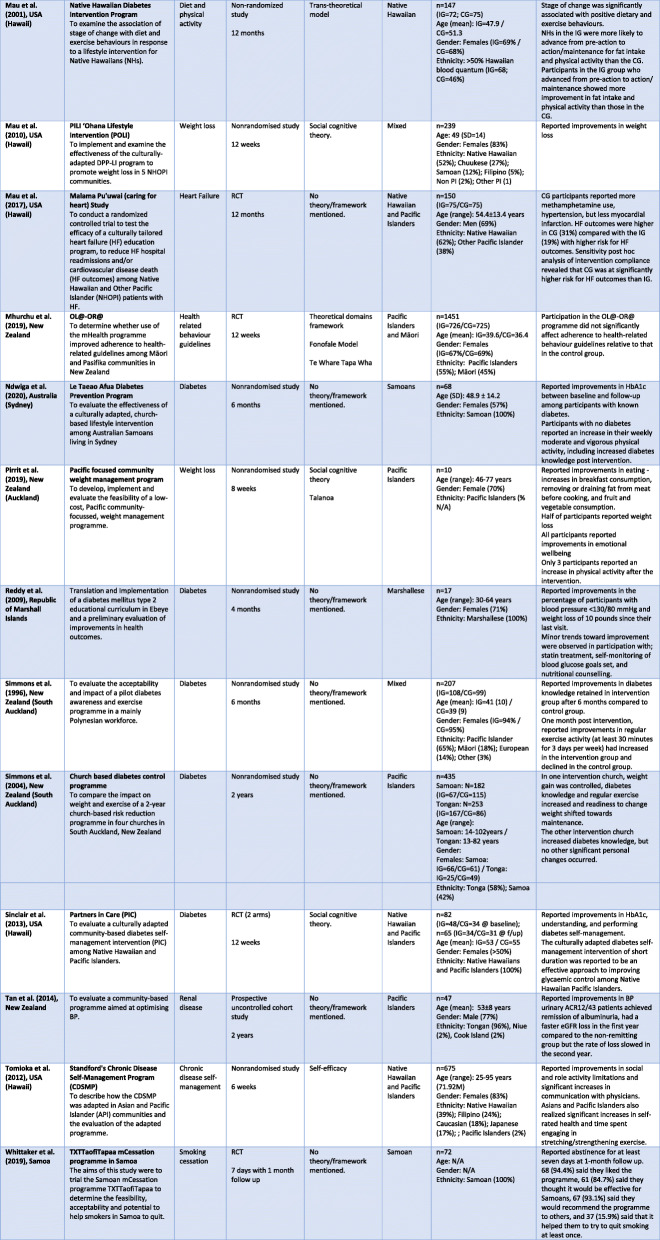
Characteristics of included studies

Among the twenty-seven studies included in the review, eleven were randomised controlled trials [44, 45, 48, 53, 55, 58, 59, 65, 66, 69] and thirteen were non randomised studies [43, 46, 47, 49, 51, 52, 56, 57, 60, 62, 64, 67, 68]. Other studies used a quasi-experimental design [63], a prospective uncontrolled cohort study [61] and a descriptive intervention study [50]. The sample sizes ranged from 10 to 675 participants, with an age range between 13 and 102 years. Across all twenty-seven studies, the samples included more than 60% female participants.

Interventions focused mainly on a single health condition or specific behaviour. Ten studies increased awareness and targeted health behaviour change in people with diabetes [45, 48, 50, 57–60, 62, 64, 68] and eight studies targeted weight loss [43, 44, 46, 47, 51, 54, 63, 67]. Others targeted chronic disease self-management [52], and health-related behaviour [66], hypertension [53, 65], smoking cessation [69], heart failure [55], renal disease [61], physical activity [56], and physical activity and nutrition combined [49]. Overall, diabetes was the most common long term condition focused in the studies.

### Theoretical frameworks

Fifteen studies stated they used a theoretical behaviour change framework in their intervention. Social cognitive theory (SCT) was most frequently used (nine studies) [43–47, 51, 53, 54, 67], with one study combining a range of theories such as participatory metatheory, the health belief model, SCT and the Pacific *talanoa* approach [67]. One study combined the theoretical domains framework with the *Fonofale* (Pacific) and *Te Whare Tapa Whā* (Māori) health models [66]. Other studies were informed by the trans-theoretical model [49], social support model [48, 57], Precede-Proceed model [58], and self-efficacy framework [52]. Various combinations of BCTs were incorporated across all 27 studies.

### Behaviour change techniques

Using the behaviour change taxonomy [11], 27 different BCTs (from a total of 93) were identified and coded across all the interventions. Each study incorporated BCTs using different combinations, ranging from three to 18. The most common BCT was instruction on how to perform a behaviour (*n* = 27), followed by social support (general) (*n* = 25) and behaviour rehearsal/practice (*n* = 21) as seen in Table 3. The two studies that did not provide social support instead focused on behaviour change solely on the individual, despite other interventions delivered to individuals incorporating social support from family and friends. Other commonly used BCTs included health consequences (*n* = 16), problem-solving / coping planning (*n* = 14), self-monitoring of outcome(s) of behaviour (*n* = 13), modelling of the behaviour (*n* = 13), and goal setting (behaviour) (*n* = 11). Each study reported changes in clinical and behavioural outcomes with the various BCTs used. Due to heterogeneity of outcome measures, a meta-analysis was not undertaken to determine the effectiveness of BCTs used in each study.

**Table 3:**
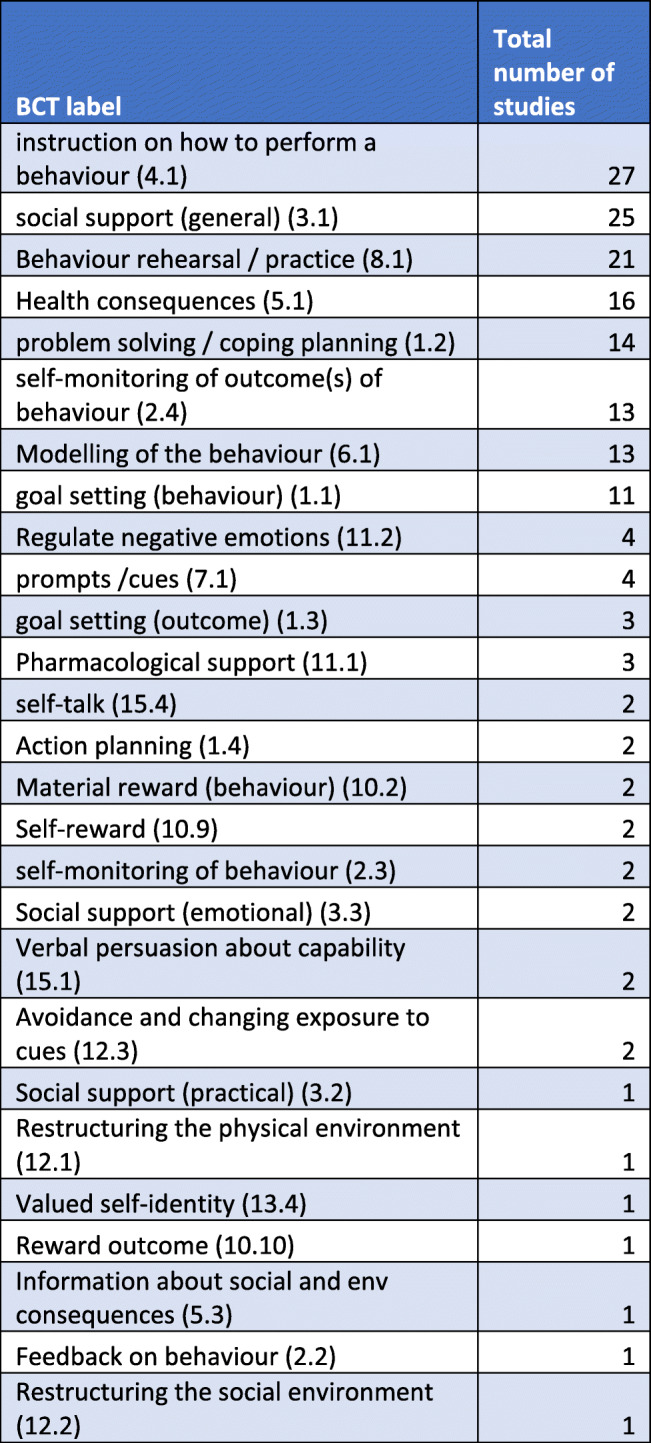
Frequency of behaviour change techniques used

### Intervention elements

All but one study [65] culturally adapted or added cultural elements to the design of their programme which closely resonated with Pacific people. Examples included cooking with local or culturally appropriate food, using *hula* (cultural dance) as a form of exercise, incorporating local language, and cultural customs during education sessions, and using a minister or *kupuna* (elder) to lead and close education sessions. One study used co-design [66] with Pacific (including Māori) communities to design the behaviour change intervention. Another study was informed by a Pacific nutrition train the trainer workshop for Pacific people living in New Zealand [67]. Thirteen studies were adapted for Native Hawaiian’s and Pacific Islanders living in Hawai’i [43–55], four were adapted for Samoans residing in American Samoa [58, 59], Australia [68] and Samoa [69], and two studies were adapted for Marshallese Islanders living in Arkansas [57] and the Marshall Islands [60]. Ten studies [43, 44, 46, 47, 53, 54, 56, 57, 67, 68] stated they used a CBPR approach where research leads partnered with Pacific communities and organisations who informed the cultural and community adaptation of programmes. While most interventions were delivered in English, seven studies offered a bilingual approach: one study offered English, Chuukese and Samoan [44], one offered English, Tongan and Samoan [62] and five offered English and Samoan [58, 59, 63, 68, 69]. To ensure fidelity of the programme once culturally adapted, only three studies mentioned research leads observed their facilitators [45, 50, 52].

Thirteen studies delivered their interventions alongside community-based organisations [43–46, 48, 49, 51–53, 56, 59–61], three were based in churches [62, 63, 68], three were delivered in participants workplaces [47, 54, 64] and one in a community location [67]. Two studies visited participants in their homes [55, 65] and two used mobile phones with one using an app [66] and the other delivering short message service (SMS) behaviour change messages to participants [69]. Three studies enabled participants to choose the location of their intervention, selecting either a church, community-based organisation, workplace or home [50, 57, 58]. Only eight studies encouraged participants to include family members or a significant other to be involved with education sessions [44, 48, 50, 55, 57, 58, 66, 68].

Eleven studies used peer health educators (PHE) [43–47, 49, 51, 54, 60, 62, 63] to facilitate education sessions, four studies used community health workers (CHW) [50, 53, 57, 59], one used a health care assistant (HCA) [65] while another study used the term community coach facilitators (CCF) [68]. Of the three studies based in churches, one used PHE [68] to deliver sessions to church members, while two [62, 63] set up health committees led by ministers wives and members within the church. These health committees then selected church members to become trained CHW. CHW and PHE were either trained by their research or project leads or undertook formal education through a tertiary institution [50, 62–64, 68]. The PHE, CHW, HCA and CCF always worked under the supervision of the research or project lead, and alongside a multidisciplinary team (nurses, physicians, nutritionists and pharmacists). A multidisciplinary approach was used in eight studies [43, 45–48, 51, 53, 65] where a CHW, PHE or HCA facilitated sessions or worked alongside health professionals (nurse specialist, physician, pharmacist, nutritionist, physiotherapist) including a *kumu hula* (hula expert). Three studies [58, 59, 65] used a treatment protocol which determined the length and frequency of visits by a CHW, PHE or HCA. When participants clinical observations were elevated (e.g. high blood pressure), the CHW, PHE or HCA would communicate their results with their physician for further clinical examination or treatment adjustments.

Eighteen studies delivered their intervention using group-based models [43–48, 51–57, 60, 62–64, 67, 68] and nine were delivered to individuals [49, 50, 55, 58, 59, 61, 65, 66, 69]. The intensity, duration, and time between intervention sessions varied. One study ran for a week [69], eight studies lasted twelve weeks [43, 45–48, 51, 53, 66], with others lasting six-eight weeks [52, 56, 57, 67], four months [60], six months [44, 64, 68], nine months [54], twelve months [49, 50, 55, 58, 59, 63, 65] and two years [61, 62]. Each session lasted between one to two and a half hours. Intervention follow-up ranged from three to 24 months. Six studies did not define how many hours each visit or education session lasted [47, 49, 50, 58, 59, 68]. Three interventions used multimedia with one utilising a diabetes educational video to compliment the physical activity sessions delivered [64], one used a DVD and workbook for participants [54], while another intervention used video as the primary mechanism lasting 10 min [56]. Despite the latter study lasting two-months, and targeting church groups and community organisations, it was unclear how many sessions participants viewed the video.

### Outcomes

Twenty-six studies reported positive outcomes and improvements in intended behaviour change measures. One study however found no significant difference between the intervention and control groups to health-related behaviours (physical activity, smoking behaviour, alcohol intake, fruit and vegetable intake) [66]. Five studies reported improvements in nutritional intake [46, 47, 49, 60, 67] and eight studies reported weight loss changes [43, 44, 46, 47, 51, 54, 60, 67]. Improvements in physical activity were reported in nine studies [46, 47, 49, 52, 56, 62–64, 68]. In one study [64], physical activity in the intervention group improved significantly compared to the control group while in another study [63], physical activity rates only improved in some participants, whereas those who reported they were sedentary at baseline remained unchanged post-intervention. One study [62] which ran two intervention and control groups (one Samoan and one Tongan church) reported weight, waist circumference and exercise improvements in only one of the intervention groups (Samoan), and not the second intervention group (Tongan) or either control groups.

Reported changes in Haemoglobin A1c (HbA1c) varied from baseline to follow up in six studies [45, 48, 50, 57, 60, 68]. One study [50] reported a more significant reduction in HbA1c for the intervention group than the control group, and three studies [45, 57, 60] reported positive changes post-intervention. Another study [48] however reported participant’s glycaemic control at six months was not significantly different from those in the control group despite initial improvements after the first three months.

Nine studies reported improvements in blood pressure [46–48, 51, 53, 58, 60, 61, 65] and six studies reported improvements in self-management and diabetes knowledge [45, 48, 60, 62, 64, 68]. Other reported improvements included self-reported health [52], self-efficacy [47, 52], smoking cessation [69], medication adherence [60], improved cholesterol levels [51], and increased primary care physician visits compared to emergency department visits [59]. Satisfaction and acceptability with the interventions were reported in four studies [52, 56, 62, 64].

## Discussion

The aim of this review was to describe behaviour change components used in interventions to improve health and effect health behaviour change among Pacific people. To our knowledge, it is the first study to highlight and describe the theoretical underpinnings and BCTs used in interventions designed to improve health among Pacific people. Twenty-seven studies met the inclusion criteria for the review. Most studies focused on diabetes and weight loss, followed by hypertension, physical activity, and smoking cessation.

An important feature to highlight was the collaborative CBPR approach used to culturally adapt interventions. When CBPR was not used, studies partnered with church-based organisations, workplaces, or local communities, evident from 1996 until 2020. An element that could strengthen partnerships and provide self-determination with future research agendas is the need to continue building research capacity and capability among Pacific communities. This ensures future research builds on existing capacity within Pacific communities rather than duplicating efforts with external research agencies. To do this, meaningful partnerships with Pacific communities using CBPR must be established, which demonstrates cultural integrity, rigour and acknowledgement of Pacific worldviews and values. Moreover, Pacific communities self-determination for Pacific research is necessary to improve health equity [14, 36] and culturally safe research practices [16, 70, 71].

Despite evidence of the impact on behaviour change, most interventions were short-term with varying study designs and little regard to sustainability. Only two studies followed up on weight maintenance after completing a three-month weight loss programme [44, 54]. Most interventions were also centred on Eurocentric theoretical frameworks, namely SCT. While studies are considered more effective when incorporating such theoretical components [10–12], only one study [67] included the Pacific *talanoa* approach as a means of allowing participants to share and exchange their knowledge, emotions and experiences. *Talanoa* (conversation, a talk, an exchange of ideas or thinking) provides a culturally appropriate approach for *‘va’* (relationships) to be established and nurtured between researchers, facilitators, and participants. The nurturing of these relationships creates a space where *talanoa* or social conversations can take place, holistically intermingling the knowledge, experiences and emotions, shared between researchers and participants [72]. *Talanoa* constitutes a culturally appropriate method which Pacific researchers have primarily used to engage with Pacific communities [34, 16].

One study [58] used co-design with Pacific and Māori communities, complimenting CBPR. Co-design empowers users to tailor interventions according to their cultural needs and context from design inception [64]. As such, participants in this study aligned their wellbeing priorities with ethnic-specific models of health and wellbeing, namely *Fonofale* [23] (Pacific) and *Te Whare Tapa Whā* (Māori) [73]. Despite co-designing a culturally tailored, lifestyle support intervention, the authors noted participation in the control and intervention groups did not affect adherence to health-related behaviour guidelines. Perhaps considerations regarding digital inclusion and the digital health literacy skills required for this intervention were overlooked, which are known equity issues for Pacific people in New Zealand [74, 75].

All studies in this review incorporated cultural adaptations and elements, and utilised BCTs of some sort. Using Michie’s behaviour change taxonomy [11], 27 BCTs (out of a possible 93) were identified and used in different combinations across all studies. The minimum number of BCTs used in a study was three, the majority being 18 techniques. Specific behaviours targeted included combinations of physical activity, healthy eating, self-management, medication adherence, problem-solving, coping, and increasing knowledge of health conditions. The most popular BCT was instruction on how to perform a behaviour (4.1), used to demonstrate culturally appropriate meals, facilitate exercise classes and provide educational sessions around the targeted health conditions and behaviours; followed by social support (unspecified) (3.1). Providing social support resonates with Pacific values, drawing strength from socio-cultural relationships within their collective contexts, such as extended family, community, and church networks [17, 22, 28].

Most studies in this review were based in the USA or its affiliate countries (American Samoa and the Marshall Islands). Only seven were from New Zealand and one from Australia and Samoa. All but two of the studies were representative of Pacific countries from Polynesia, with another from Micronesia (Marshall Islands). Importantly, there were no studies from Melanesian countries that met our search criteria, despite evidence showing they too experience high rates of long-term conditions [76]. Pacific people throughout the diaspora are diverse with different contexts and cultural constructs, language, migration histories, constitutional ties and health needs [16, 17]. Even though Pacific people share many commonalities, they are not a homogenous group.

A key component for more than half the studies, which needs to be acknowledged, was the utilisation of Pacific health workers (CHW, PHE, HCA) from participants own communities. Even if they were not leading intervention components, they worked within a multidisciplinary team who provided supervision and support. In one study [53], PHE worked alongside a *kumu hula* (hula expert), who delivered hula lessons while PHE focused on the education modules. Another study [62] found church members were more connected to the facilitators from their church than those who were not, which is essential to consider. Few differences were found between the different roles (CHW, PHE, HCA) as all were required to undertake training before working with participants. Studies that used Pacific health workers provided an important link between academia and the community, assisting with the cultural adaptation of programmes and supporting participants through behaviour change. Bilingual programmes may be more effective by overcoming language barriers, especially when interventions are delivered predominately in English. Pacific health workers’ added value was their ability to connect all the elements within each study (i.e. language, ethnicity, BCT, facilitating education sessions) effectively enhancing social support and promoting positive behaviour change outcomes. Building capacity among Pacific communities to self-determine their research aspirations is essential for community-led and owned research [34, 70, 71].

A positive outcome, not well represented in the literature, was the minister’s wives role as facilitators within a church setting. Minister’s wives were key to establishing health committees and facilitating nutrition and exercise programmes. Policy change was also possible within the church context. One church [62] created policies to incorporate nutritional guidelines for the congregation and another [63] invested in five members to become key facilitators for the nutrition and physical activity components. Churches can establish themselves in a way where health interventions could be mobilised with the right support structures in place. Churches have long been viewed as an extension of the family, preserving traditions and cultures, and mediating between the community and broader society [22]. As such, it is not surprising positive behaviour change outcomes were reported within this faith-based setting.

Although most interventions were delivered alongside community-based organisations, alternative sites such as workplaces, churches, homes, and the use of multimedia and mobile phones offer non-traditional approaches that are also acceptable for Pacific communities. Incorporating traditional cultural art forms such as *hula* also draw on the cultural nuances that have resonated with Pacific communities for centuries. The wide age range of 13 and 102 years was only evident in the group delivered interventions based in churches. Though not the focus of this review, including young people in interventions alongside their parents and grandparents, enables an intergenerational approach to improving health and wellbeing within Pacific families [77]. Furthermore, evidence suggests that exercise among older people is protective against fall-related fractures [78]. More studies reporting on the long-term effects of BCTs would be beneficial to determine factors supporting sustained behaviour change among Pacific populations.

### Limitations

A key limitation of this review is due to heterogeneity of participants, types of interventions and outcomes, a meta-analysis was not completed. Therefore, conclusions cannot be drawn on the effectiveness of behaviour change interventions among Pacific people. All eligible studies focused on Pacific populations from Polynesia living in the USA, American Samoa, and New Zealand. One study focused on the Marshall Islands, Australia, and Samoa, which limits the generalisability of findings. This review was also limited to studies with more than 40% of the study population identified as Pacific. This would have excluded other studies that included a smaller sample of Pacific people.

## Conclusion

This review provides new and important insights into potential elements and components when designing behaviour change interventions for Pacific people. It also highlights the paucity of literature available for Pacific communities living outside of the USA.

Future behaviour change research with Pacific communities should be community created and owned, culturally anchored, and centred on a collective approach. Culturally relevant interventions are essential for uptake and maintenance of behaviour change programmes. CBPR provides a useful framework to ensure interventions are culturally grounded, which is vital for the uptake and maintenance of behaviour change when programmes initially intended for non-Pacific populations are adapted. Framing behaviour change from the context of Pacific cultural values becomes an integral part of this process. Moreover, negotiating these spaces through *talanoa* and understanding how the physical, social, and spiritual elements are intrinsically linked to the sustenance of Pacific people’s health and wellbeing is critical [28]. While interventions can include common cultural elements; approaches need to be contextualised to each Pacific Island community.

Community centred aspirations determined by Pacific communities is fundamental to ensuring the health outcomes measured by interventions, are elements that are relevant and applicable to their lived realities and worldviews. Future research needs to invest in building research capacity within Pacific communities, centering self-determining research agendas, and findings to be led and owned by communities. This recognises Pacific communities are more than programme facilitators.

## Supplementary Information

**Additional file 1.** PRISMA study selection process

## Data Availability

The datasets used and analysed in the current study are available from the corresponding author on reasonable request.

## Abbreviations

BCT: Behavior change techniques
CBPR: Community based participatory research
CCF: Community coach facilitators
CHW: Community health workers
HbA1c: Haemoglobin A1c
HCA: Health care assistant
PHE: Peer health educators
POP: Pili Ohana Project
PRISMA: Preferred Reporting Items for Systematic Reviews and Meta-Analyses
SCT: Social cognitive theory
USA: United States of America
